# Potential Canonical *Wnt* Pathway Activation in High-Grade Astrocytomas

**DOI:** 10.1100/2012/697313

**Published:** 2012-08-02

**Authors:** Rebecca Schüle, Christine Dictus, Benito Campos, Feng Wan, Jörg Felsberg, Rezvan Ahmadi, Franz-Simon Centner, Niels Grabe, Guido Reifenberger, Justo L. Bermejo, Andreas Unterberg, Christel Herold-Mende

**Affiliations:** ^1^Division of Neurosurgical Research, Department of Neurosurgery, University Hospital Heidelberg, INF 400, 69120 Heidelberg, Germany; ^2^Hertie-Institute for Clinical Brain Research, University of Tuebingen, 72076 Tuebingen, Germany; ^3^Department of Neurosurgery, Tongji Hospital, Huazhong University of Science & Technology, 430030 Wuhan, China; ^4^Department of Neuropathology, University of Düsseldorf, 40225 Düsseldorf, Germany; ^5^Institute of Medical Biometry and Informatics, Hamamatsu Tissue Imaging and Analysis (TIGA) Centre, University of Heidelberg, 69120 Heidelberg, Germany; ^6^Institute of Medical Biometry and Informatics, University of Heidelberg, 69120 Heidelberg, Germany; ^7^Division of Molecular Genetic Epidemiology, German Cancer Research Centre, 69120 Heidelberg, Germany

## Abstract

Aberrant *wnt* pathway activation through cytoplasmic stabilization of **β**-catenin is crucial for the development of various human malignancies. In gliomagenesis, the role of canonical (i.e., **β**-catenin-dependent) signalling is largely unknown. Here, we studied canonical *wnt* pathway activation in 15 short-term cultures from high-grade gliomas and potential pathomechanisms leading to cytoplasmic **β**-catenin accumulation. Furthermore, we assessed the prognostic relevance of **β**-catenin expression in a tissue microarray comprising 283 astrocytomas. Expression of **β**-catenin, its transcriptional cofactors TCF-1 and TCF-4 as well as GSK-3**β** and APC, constituents of the **β**-catenin degradation complex was confirmed by RT-PCR in all cultures. A cytoplasmic **β**-catenin pool was detectable in 13/15 cultures leading to some transcriptional activity assessed by luciferase reporter gene assay in 8/13. Unlike other malignancies, characteristic mutations of **β**-catenin and APC leading to cytoplasmic stabilization of **β**-catenin were excluded by direct sequencing or protein truncation test. In patient tissues, **β**-catenin expression was directly and its degradation product's (**β**-catenin-P654) expression was inversely correlated with WHO grade. Increased **β**-catenin expression and low **β**-catenin-P654 expression were associated with shorter survival. Altogether, we report on potential canonical *wnt* pathway activation in high-grade gliomas and demonstrate that **β**-catenin expression in astrocytomas is associated with increased malignancy and adverse outcome.

## 1. Introduction

Deregulation of cell cycle control and activation of growth factor-associated pathways are main mechanisms in the development of astrocytic gliomas [1]. As crosstalks between growth factor pathways and the *wnt* signalling cascade have been uncovered recently [2–4], this study aimed at investigating the role of *wnt* pathway activation in glioma pathogenesis.

Activating mutations in this pathway contribute to neoplastic transformation in various human tissues. The common hallmark of these mutations is the nuclear accumulation of *β*-catenin, the main effector of the canonical (i.e., *β*-catenin-dependent) *wnt* pathway. In the absence of *wnt* ligands, *β*-catenin is regulated by a multiprotein complex comprising at least four interacting proteins: the tumour suppressor APC, the scaffolding protein axin, and the phosphokinases GSK-3*β* and CK1 [5]. Upon ligand binding, the degradation complex is destabilized, allowing for cytoplasmic *β*-catenin accumulation with subsequent nuclear translocation. In the nucleus, *β*-catenin acts as an essential cofactor in the context of cell cycle regulation, differentiation, migration, proliferation, and survival [6].

In many cancers, among which colorectal cancers are probably the best studied example, aberrant *wnt* pathway activation is mimicked by cytoplasmic *β*-catenin stabilization [7–11]. In this study, we report on cytoplasmic accumulation of *β*-catenin in high-grade gliomas leading to some transcriptional *wnt *activation. Concomitantly, we exclude characteristic activating mutations in the APC or *β*-catenin gene as a cause for cytoplasmic *β*-catenin stabilization, as they frequently occur in other tumour entities. Further, we describe an increased *β*-catenin expression in astrocytomas that correlates with malignancy grade and expression of the stemness marker nestin. In contrast, expression of *β*-catenin-P654, which can be regarded as inactivated degradation product of *β*-catenin [12], decreases with tumour malignancy. Finally, we report that both increased *β*-catenin and decreased *β*-catenin-P654 expression are associated with a poor patient outcome. Altogether, our data point to a potential canonical *wnt* pathway activation in astrocytic high-grade gliomas.

## 2. Materials and Methods

### 2.1. Cell Culture Conditions

Short-term glioma cultures (*n* = 16) were established as described [13]. The cell lines SW480, A172, and U373 were obtained from the cell culture bank of the German Cancer Research Centre (Heidelberg, Germany). Cells grew as adherent monolayers in DMEM medium containing 10% FCS as described elsewhere [13].

### 2.2. RNA Isolation and RT-PCR

Total RNA isolation from glioma cultures and RT-PCR reactions to determine mRNA expression of *β*-catenin, APC, GSK-3*β*, TCF1, TCF4, and GAPDH was performed according to the manufacturer's instructions using 35 amplification cycles (Qiagen RNeasy; Qiagen, Hilden, Germany, and Geneamp; Applied Biosystems, Weiterstadt, Germany). Primers are summarized in Table 1.

### 2.3. Preparation of Whole Cell Lysates and Subcellular Fractionation

For preparation of whole cell lysates, cells grown to 80% confluency were washed twice with PBS and lysed in CSK buffer (150 mM NaCl, 300 mM Sucrose, 10 mM Pipes pH 6.8, 3 mM MgCl_2_, 0.5% Triton X-100, 1x Complete Protease Inhibitor Cocktail (Roche, Mannheim, Germany), 20 *μ*M DNAse I). The suspension was centrifuged for 10 min at 14.000 rpm and 4°C and the supernatant stored at −80°C. Protein concentration was quantified colorimetrically using the DC Protein Assay (BioRad Laboratories, Hercules, CA, USA).

For subcellular fractionation, cells were grown to 80% confluency, washed twice in PBS, and lysed in homogenization buffer (10 mM Hepes pH 7.4, 1 mM EDTA, 240 mM Sucrose, 1x Complete Protease Inhibitor Cocktail). Following mechanical disruption of the cells, the lysate was centrifuged for 10 min at 600 g and 4°C, the pellet resuspended in homogenization buffer and centrifuged again. The collected supernatants of both centrifugation steps containing the combined membranous and cytoplasmic fraction were ultracentrifuged for 1 hour at 100.000 g and 4°C. The supernatant containing the cytoplasmic fraction was stored at −20°C. 

### 2.4. Immunoblotting

Equal amounts of protein were separated on 10% polyacrylamide SDS gels, blotted onto PVDF Hybond P membranes (Amersham Biosciences, Freiburg, Germany), and detected with mouse anti-*β*-catenin (BD Transduction Laboratories; San Jose, CA, USA) and HRP-coupled anti-mouse antibodies (Vector Laboratories; Burlingame, CA, USA). Western Blot bands were quantified using the software ImageJ (http://rsbweb.nih.gov/ij/).

### 2.5. Cell Transfection and TOPFLASH/FOPFLASH Reporter Gene Assay

Cells were transfected using Effectene and Superfect transfection systems (Qiagen, Hilden, Germany) following the manufacturer's instructions and harvested 24 h–48 h after transfection. Reporter plasmids (pTOPFLASH, pFOPFLASH) were a gift from H. Clevers (Hubrecht Institute, Utrecht, The Netherlands). Cells were transfected with either pTOPFLASH or pFOPFLASH and cotransfected with the *β*-galactosidase expression plasmid pCMV-beta (Clontech; Mountain View, CA, USA) to provide an internal control for the normalization of all data for transfection efficiency, cell viability, and cell number. pTOPFLASH contained the firefly luciferase ORF under control of a *β*-catenin/TCF regulated promoter. In the presence of endogenous nuclear *β*-catenin, luciferase transcription was activated by *β*-catenin/TCF. In pFOPFLASH, the TCF binding motif was defective. Cell lysis and luciferase and *β*-galactosidase detection were performed using the dual-light system (Applied Biosystems; Foster City, CA, USA) and the luminometer Lumat LB9507 (Berthold; Bad Wildbach, Germany). 

### 2.6. DNA Extraction, PCR Amplification and Sequencing

DNA was extracted from glioma cultures using the QiAmp DNA mini Kit (Qiagen; Hilden, Germany) following the manufacturer's instructions. Codons 957 to 1513 of the APC coding sequence, containing the mutations cluster region as well as mutational hotspots at codon 1061 and codon 1309, were amplified (Geneamp; Applied Biosystems, Weiterstadt, Germany). *β*-catenin exon 3 was amplified including exon-intron boundaries. Sequencing was performed using the ABI PRISM BigDye Terminator v3.1 Cycle Sequencing Kit (Applied Biosystems; Foster City, CA, USA). Sequencing products were resolved on an ABI 3100 automated sequencer (Applied Biosystems; Foster City, CA, USA) and analyzed using Staden Package [14].

### 2.7. APC Protein Truncation Test

After reverse transcription of RNA extracted from glioma cells, APC cDNA was amplified in five segments. Segment 1 contained exons 2–15, segments 2–5 spanned the large exon 16. Each forward primer contained a T7 promoter, Kozak sequence, and start codon, to allow subsequent *in vitro* transcription. For *in vitro* translation, biotinylated lysine tRNA was used. The resulting protein fragments were separated by SDS-PAGE, transferred to a PVDF membrane, and detected using streptavidin-peroxidase. 

### 2.8. Tissue Microarray

The tissue microarray (TMA) consisted of formalin-fixed, paraffin-embedded tissue samples derived from 283 patients operated at the Department of Neurosurgery at Heidelberg University, Germany, for astrocytomas of WHO grade II, III, or IV. Informed consent was obtained from each patient according to the research proposals approved by the Institutional Review Board at Heidelberg Medical Faculty. Median follow-up time was 11.0 years (±4.4 years). Overall survival was calculated from the first date of diagnosis till the time of death or the provisional endpoint of the study (November 30th 2009). Patient characteristics are reported in Table 2.

### 2.9. Immunohistochemistry

Primary antibodies used for immunohistochemistry were mouse-monoclonal anti-*β*-catenin (Zytomed Systems; Berlin, Germany) and mouse-monoclonal anti-*β*-catenin-P654 (*β*-catenin phosphorylated at tyrosine 654, Biodesign; Maine, USA). Antigen retrieval was performed in a water bath at 100°C for 20 min using antigen retrieval buffers at pH 9.9 (DAKO; Hamburg, Germany). Prior to TMA staining specificity of primary antibodies was ensured using appropriate isotype controls (all acris; Hiddenhausen, Germany), and antigen expression was evaluated in a small study sample comprising astrocytoma tissues of WHO grades II–IV (*n* = 20 –30) to establish suitable antigen grading categories based on antigen expression variability (microscope Olympus BX50, Olympus, Hamburg, Germany; software Cell Imaging, Olympus, Hamburg, Germany). Staining was performed as previously described [15]. Each biopsy on the TMA slides was evaluated at 20x magnification after whole-slide imaging digitized with the Hamamatsu NanoZoomer Digital Pathology slide scanner (Hamamatsu Photonics, Japan). Staining was semiquantitatively graded from 0 to 4 according to the percentage of positive cells covering the whole tissue spot: *β*-catenin: 0% = 0; >0–10% = 1; >10–50% = 2; >50–80% = 3; >80–100% = 4; *β*-catenin-P654: 0% = 0; >0–1% = 1; >1–5% = 2; >5–10% = 3; >10% = 4. Average staining results from all biopsies of each individual patient were taken as final result. For statistical analysis, intermediate staining frequencies were used as cutoffs to group patients into “low expression” and “high expression” cohorts (*β*-catenin: ≤50% versus >50%; *β*-catenin-P654: ≤5% versus >5%). For immunofluorescence staining, primary antibodies (mousemonoclonal anti-*β*-catenin (BD Transduction Laboratories; San Jose, CA, USA); rabbit-polyclonal anti-nestin (Millipore, Schwalbach, Germany)) were visualized with fluorophore-conjugated secondary antibodies (anti-mouse Alexa Fluor 488, anti-rabbit Alexa Fluor 555; all Invitrogen, Karlsruhe, Germany). 4′,6-Diamidino-2-phenylindole (DAPI) was used for nuclear counterstaining.

### 2.10. CTNNB1 Knockdown

For knockdown experiments, commercially available plasmids harboring CTNNB1 shRNA or a scrambled control were used following the manufacturer's instructions (BioCat, Heidelberg, Germany). Knockdown was performed on an additional cell line derived from a primary glioblastoma (NCH421k), which was chosen for its high expression of nestin [16].

### 2.11. Quantitative PCR

RNA isolation was performed using the RNeasy Mini Kit (Quiagen, Hilden, Germany) according to the manufacturer's instructions. An endogenous housekeeping gene (*PITPNB*) was used for internal normalization. The following primers were used in our study: *CTNNB1* mRNA forward primer 5′-GCTTTCAGTTGAGCTGACCA-3′; *CTNNB1* mRNA reverse primer 5′-CAAGTCCAAGATCAGCAGTCTC-3′; *NESTIN* mRNA forward primer 5′-TGCGGGCTACTGAAAAGTTC-3′; *NESTIN* mRNA reverse primer 5′-TGTAGGCCCTGTTTCTCCTG-3′; *PITPNB* forward primer 5′-CGAGACTCAGAAAGAACTAGAAACAA-3′; *PITPNB* reverse primer 5′-TGACCCTACAGGGGACTCAT-3′. 

### 2.12. Statistical Analysis

Recurrent tumours and patients with incomplete clinical followup were excluded from the survival analysis. Association between overall survival (OS) and staining frequencies of individual antigens was calculated using the log-rank test and presented as Kaplan-Meier plots. In multivariate Cox regression analyses, hazard ratios were adjusted for the influence of prognostic factors on OS, that is, WHO grade, patient age at diagnosis, and extent of tumour resection. All calculations were performed using the statistical software environment R, version 2.4.1 (http://www.r-project.org/). *P* values ≤0.05 were considered statistically significant.

## 3. Results

### 3.1. Key Players of the *Wnt* Pathway Are Expressed in High-Grade Glioma

Presence and functional significance of *wnt*/*β*-catenin signalling in high-grade gliomas were studied in short-term cell cultures established from ten glioblastomas WHO grade IV, two gliosarcomas WHO grade IV (NCH37, NCH57), and one anaplastic oligodendroglioma WHO grade III (NCH262). The established glioblastoma cell lines A172 and U373 were included in the study as well as the human colon adenocarcinoma cell line SW480, which served as positive control.

**Figure 1:**
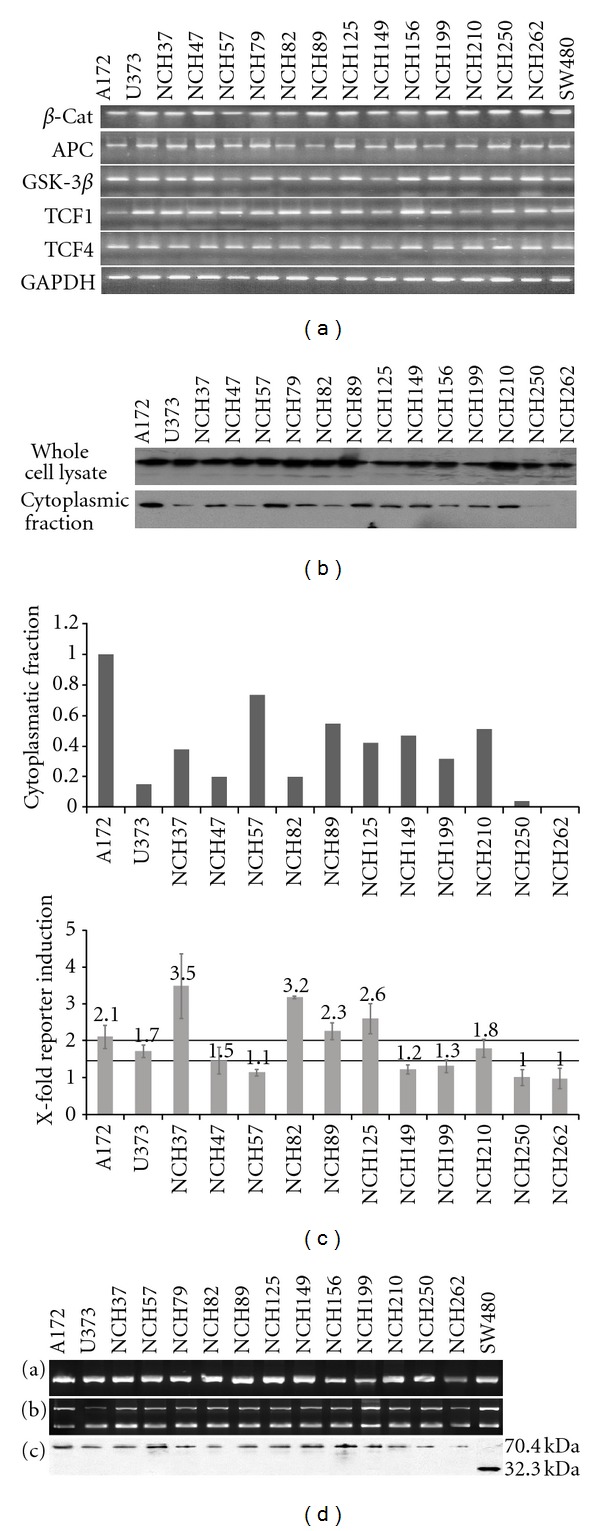
(a) Gel electrophoresis showing expression of key players of the canonical *wnt* signalling pathway as assessed by RT-PCR. (b) (upper rows) Representative western blot analysis of 3 independent experiments displaying *β*-catenin expression in whole cell lysates and corresponding cytoplasmic fractions of high-grade glioma cultures. (lower graph) Quantification of the cytoplasmatic *β*-catenin fractions shown in (b). Expression levels were normalized to A172. (c) Transcriptional activity of *β*-catenin in glioma cultures as assessed by TOPFLASH/FOPFLASH reporter gene assay. Ordinate shows x-fold induction of the luciferase reporter gene in comparison to the control vector (FOPFLASH). Horizontal lines mark 1.5-fold and 2-fold reporter induction, respectively. Data indicate mean values of at least 3 independent experiments plus SD. (d) For theAPC truncation test, APC cDNA was amplified in five fragments and subsequently transcribed and translated *in vitro*. Products were separated by SDS-PAGE, blotted on a PVDF membrane, and stained with streptavidin peroxidase. No truncating mutations were found in any of the glioma cultures. Here, cDNA amplification (a), *in vitro* transcription (b) and *in vitro* translation (c) of fragment 3 that contains the mutation cluster region are shown. The colon cancer cell line SW480, which contains a homozygous truncating mutation at codon 1338, served as positive control. The molecular weight of the resulting protein fragment in this cell line is thereby reduced from about 70 kDa (full-length fragment 3) to 32 kDa.

Expression of key players of the *wnt* pathway was examined by RT-PCR (Figure 1(a)). We found *β*-catenin to be expressed in all 15 glioma cultures analyzed. Expression of APC and GSK-3*β*, two members of the *β*-catenin degradation complex [17], and TCF1 and TCF4, two nuclear interaction partners of *β*-catenin [17, 18], was confirmed as well. Thus, we assumed that our cultures expressed relevant key players necessary for *wnt*/*β*-catenin signalling.

### 3.2. A Cytoplasmic Pool of *β*-Catenin Is Present in Most High-Grade Gliomas

**Figure 2:**
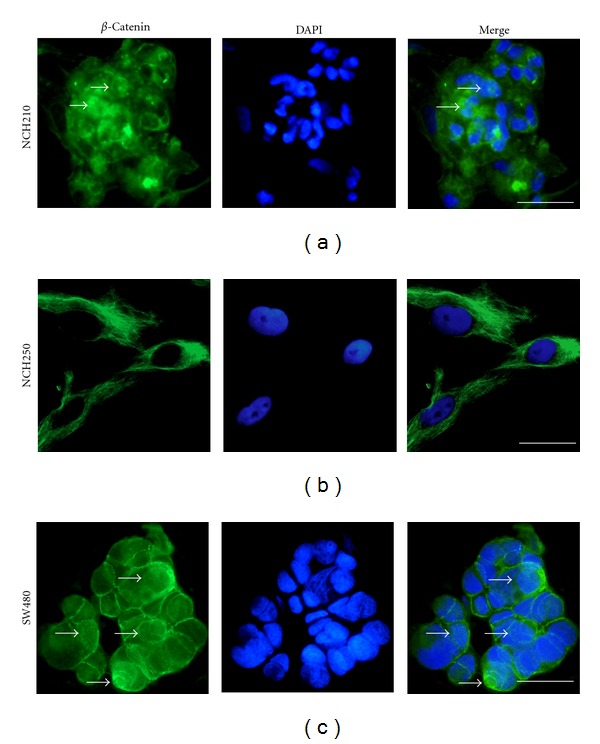
Immunofluorescent staining depicting *β*-catenin expression patterns in glioma cultures with no (NCH250 (b)) and moderately increased (NCH210 (a)) transcriptional activity and in the colon carcinoma cell line SW480 (c) known for its pronounced transcriptional activity. A preponderance of cytoplasmic and membranous rather than nuclear localization of *β*-catenin was confirmed in all cell lines analyzed. However, nuclear *β*-catenin levels (closed arrows) as a prerequisite for transcriptional activation were detected in single cells in NCH210 (a) and were relatively higher in SW480 (c). Scale bars represent 100 *μ*m (a) and 50 *μ*m (b and c), respectively.

Since aberrant *β*-catenin signalling is reflected by the cytoplasmic accumulation of the protein, we further explored a potential intracellular *β*-catenin pool. Indeed, *β*-catenin was detected by immunofluorescent staining with a predominant cytoplasmic and membranous staining pattern in glioma cultures (NCH210, NCH250; Figures 2(a) and 2(b)) and enhanced nuclear staining in the SW480 cell line (Figure 2(c)) and by western blot in whole cell lysates of all 15 glioma cultures examined (Figure 1(b), upper row). To differentiate membranebound from uncomplexed cytoplasmic *β*-catenin, subcellular fractions of glioma cell lysates were subjected to western blot analysis (Figure 1(b), lower row and graph). Cytoplasmic *β*-catenin was detected in all but one glioblastoma (11/12) and in the two gliosarcoma cultures, whereas *β*-catenin was not present in the cytoplasm of the anaplastic oligodendroglioma analyzed (NCH262). Hence, the majority of our cultures featured an aberrant cytoplasmic *β*-catenin pool.

### 3.3. *β*-Catenin Is Transcriptionally Active in High-Grade Gliomas

*Wnt/β*-catenin signalling depends on the cytoplasmic stabilization of *β*-catenin. In order to exert its function as a transcription factor, however, *β*-catenin must first translocate to the nucleus. To investigate whether the aberrant cytoplasmic accumulation of *β*-catenin was accompanied by increased transcriptional activation we performed a luciferase reporter gene assay to directly assess transcriptional activity of the *β*-catenin/TCF complex. Glioma cultures were transiently transfected with reporter plasmids containing an intact TCF binding motif (pTOPFLASH) or a defective TCF binding motif (pFOPFLASH) (Figure 1(c)). 5/13 cultures examined showed an over 2-fold increase in reporter induction in relation to induction by the defective pFOPFLASHreporter and 8/13 cultures showed an at least 1.5-fold increase in reporter induction. Although we did not observe a direct correlation of cytoplasmic *β*-catenin levels and reporter induction (Figure 1(b)), all these cultures showed a cytoplasmic *β*-catenin pool in western blot analysis, whereas the 2 glioma cultures that lacked a substantial amount of cytoplasmic *β*-catenin (NCH250, NCH262) did not show transcriptional activity in the luciferase reporter gene assay. In the remaining 3/13 cultures only a borderline increase in reporter induction was measured despite cytoplasmic *β*-catenin accumulation. Altogether, there is evidence for *wnt *pathway activation by cytoplasmic stabilization of *β*-catenin in high-grade glioma, even though cytoplasmic accumulation was not necessarily succeeded by increased transcriptional activity in all glioma cell lines.

### 3.4. Common Activating *Wnt* Pathway Mutations Are Not Responsible for Aberrant Cytoplasmic Accumulation of**** *β*-Catenin

Activating mutations in the course of the *wnt* pathway have been described in various tumours [8, 10, 19]. Loss of function mutations in the tumour suppressor APC are by far the most common and can be found in a high percentage of colorectal cancers [20]. About 50% of the known mutations are located in the mutation cluster region (codon 1286–1513) that is coded by exon 16. We sequenced the gene region between codon 957 and 1513 that contains both the mutation cluster region and the *β*-catenin binding site of APC. No APC mutations were found in any of the 13 glioma cultures that could have contributed to the accumulation of cytoplasmic *β*-catenin. Since ~95% of known APC mutations introduce a preterminal stop codon and lead to formation of a truncated protein, we further employed a protein truncation test to screen for additional APC mutations [20]. APC cDNA was amplified in five fragments and transcribed and translated *in vitro*. The size of the generated protein was then examined by SDS-PAGE (Figure 1(d)). In line with our previous results, no truncating APC mutations were found. Having ruled out direct APC mutations, we focused on potential APC-activating mutations of the *β*-catenin gene CTNNB1, which have been identified as a frequent event in various tumour entities [21]. Since almost all known oncogenic CTNNB1 mutations are located in exon 3 that codes for the N-terminal regulatory domain of the *β*-catenin protein, we sequenced CTNNB1 exon 3 including the adjacent splice sites. Again, no mutations were identified in any of the 13 glioma cultures. Thus, we were able to rule out that common mutations in the APC or *β*-catenin gene have caused the aberrant cytoplasmic accumulation of *β*-catenin.

### 3.5. WHO Grade-Dependent and Clinically Relevant Expression of *β*-Catenin and Phosphorylated *β*-Catenin-P654

**Figure 3:**
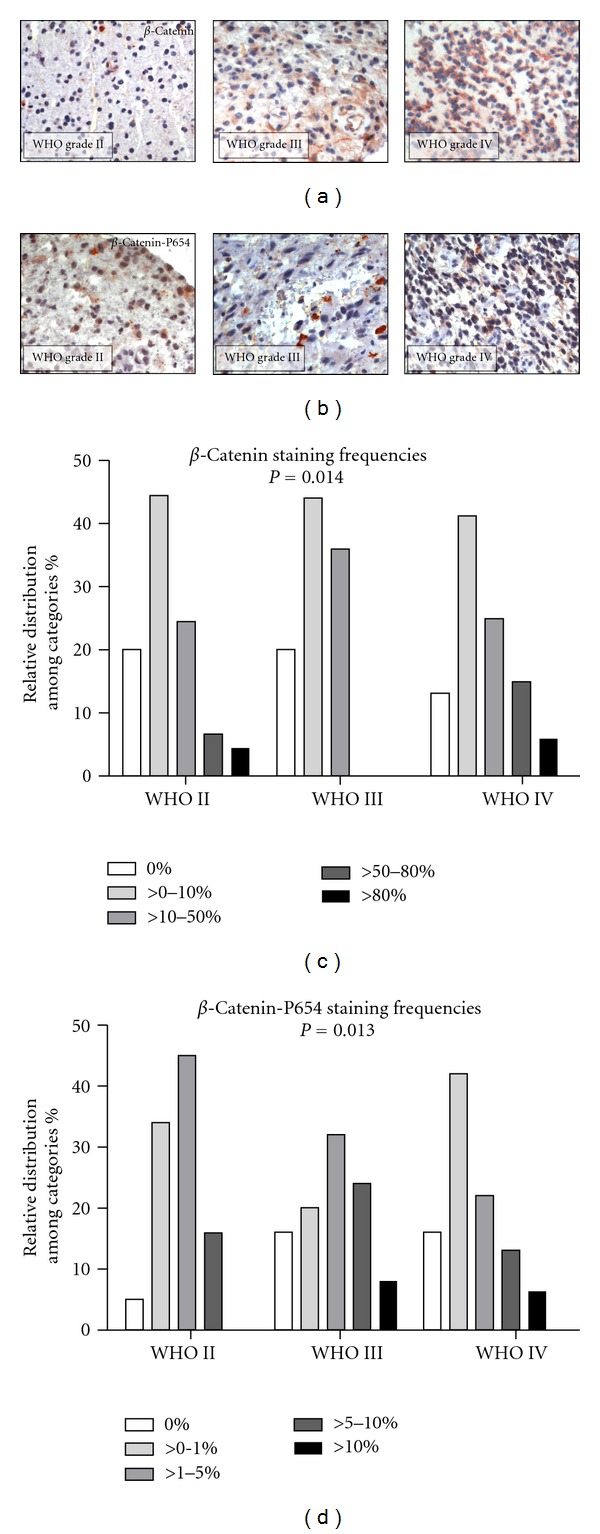
(a and b) Immunoreactivity of *β*-catenin (upper row) and *β*-catenin-P654 (lower row) in astrocytic gliomas of WHO grade II (left), WHO grade III (middle), and WHO gradeIV (right). Scale bars represent 100 *μ*m. (c and d) Note that *β*-catenin immunoreactivity was significantly augmented with increasing WHO grade (c) while *β*-catenin-P654 expression significantly decreased in high-grade tumours (d). Columns indicate categories of staining frequencies.

**Figure 4:**
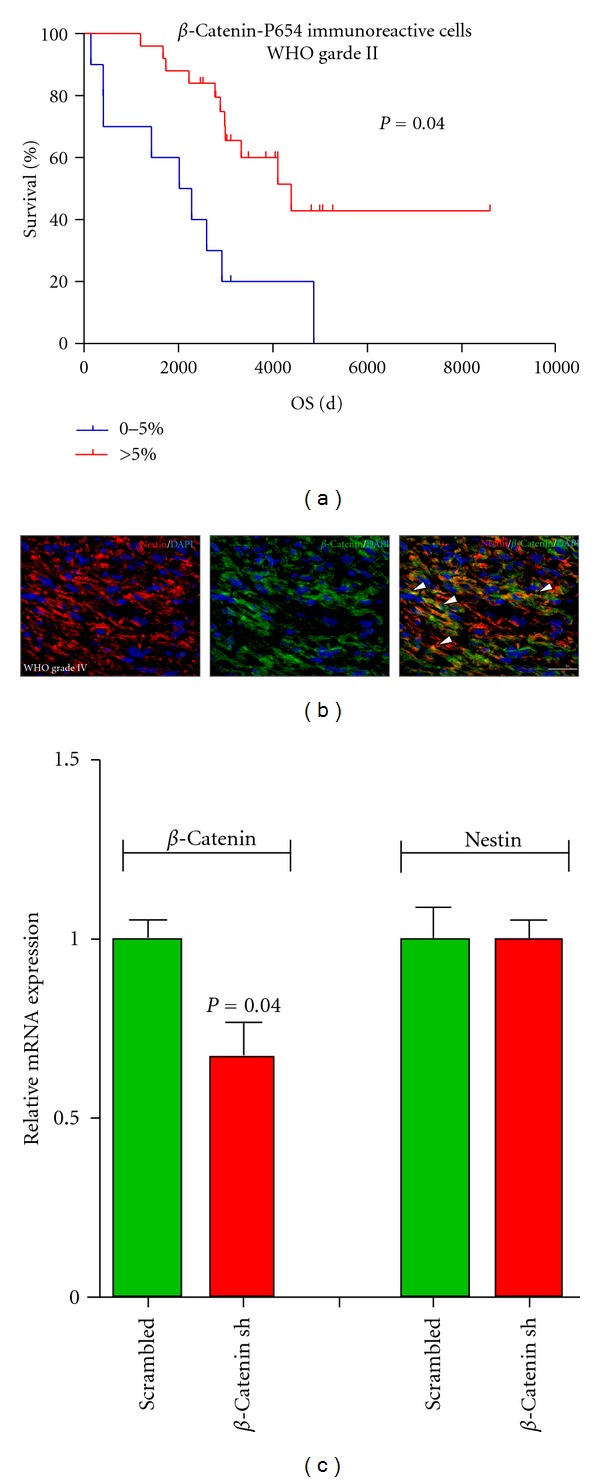
(a) Kaplan-Meier plot illustrating the correlation of patient outcome with *β*-catenin-P654 expression levels in the subgroup of low-grade astrocytomas. Expression of *β*-catenin-P654 is directly associated with a prolonged overall survival (OS). *P* value represents multivariate associations of staining frequencies with OS. (b) Immunofluorescence staining showing colocalization of the stem cell-associated filament protein nestin (red) and *β*-catenin (green). DAPI (blue) was used for nuclear counterstaining. Representative staining is shown in a primary glioblastoma tissue (white arrow heads represent double-positive cells; scale bars represent 150 *μ*m). (c) Graphs show *β*-catenin and nestin mRNA expression in NCH421k cells infected with scrambled control or a shRNA directed against *β*-catenin. Data are shown as mean values ± SEM of four technical replicates.

To elucidate whether aberrant cytoplasmic accumulation of *β*-catenin influences the clinical behaviour of gliomas, we performed an expression analysis for *β*-catenin and its degradation product phosphorylated at tyrosine P654 (referred to as *β*-catenin-P654) on a tissue microarray comprising samples of 283 astrocytomas of WHO grade II, III, or IV (Figures 3(a) and 3(b)). *β*-Catenin expression significantly correlated with higher WHO grade (rho = 0.16, *P* = 0.014) showing the strongest expression in glioblastomas WHO grade IV (Figure 3(c)). At the same time, we observed a significant WHO grade-dependent decrease in the expression of *β*-catenin-P654 (rho = −0.16, *P* = 0.013) (Figure 3(d)). In astrocytomas of WHO grade II to IV, univariate survival analysis revealed a significant association of increased *β*-catenin expression with OS (*P* = 0.05, HR 1.43 1.01–2.02). Moreover, increased expression of *β*-catenin-P654 was associated with better OS (*P* < 0.01, HR 0.63 0.48–0.83). An adjustment for known prognostic confounders revealed that the association of *β*-catenin/*β*-catenin-P654 and survival were in part attributable to the relationship of immunoreactivity with WHO grade (*P* = 0.18, HR 1.49 0.83–2.69; *P* = 0.27, HR 0.85 0.63–1.14). Nevertheless, in astrocytomas of WHO grade II, we still found a significant association between increased expression of *β*-catenin-P654 and prolonged OS (*P* = 0.04, HR0.37 0.14–0.96; Figure 4(a)). *P*-values and HRs are summarized in Table 3.

### 3.6. *β*-Catenin Expression Significantly Correlates with the Expression of the Stem Cell-Associated Protein Nestin

Given the fact that, in other tumor entities, cytoplasmic accumulation of *β*-catenin is involved in tumor proliferation and/or tumor differentiation, we correlated the expression of *β*-catenin and *β*-catenin-P654 with a previously compiled data set generated on the same TMA. This included expression of the proliferation marker Ki-67 as well as the stem cell-associated intermediate filament protein nestin [15]. Whereas Ki-67 was not correlated with any of the two *β*-catenin forms, we found a significant association between *β*-catenin and nestin expression in WHO grade IV tumours (rho = 0.23, *P* = 0.002), which was confirmed by demonstrating colocalization of *β*-catenin and nestin by double immunofluorescence staining (Figure 4(b)). However, reduction of *β*-catenin levels *in vitro* by shRNA did not affect nestin expression (Figure 4(c)), suggesting that *β*-catenin is not directly involved in the transcriptional regulation of nestin in our cell lines.

## 4. Discussion

Aberrant *wnt* pathway activation through cytoplasmic stabilization of *β*-catenin is a key event in the development of numerous human malignancies. However, data on canonical *wnt* signalling in high-grade gliomas are sparse [22, 23]. In this study, cytoplasmic *β*-catenin was identified as a frequent event in high-grade glioma cultures. In accordance with the common concept that cytoplasmic stabilization of *β*-catenin is the crucial step in regulating the canonical *wnt* signal, only tumors with a cytoplasmic pool of *β*-catenin showed increased transcriptional *β*-catenin/TCF-dependent activity in our study. However, presence of a cytoplasmic *β*-catenin pool did not always lead to transcriptional activity of *β*-catenin in the nucleus. Presumably, the balance of cytoplasmic versus nuclear retention factors of *β*-catenin as well as the presence of nuclear coactivators and corepressors might also modulate downstream *β*-catenin/TCF signalling in gliomas. 

Interestingly, common *wnt* pathway mutations that cause cancer in various other tissues could be excluded as potential causes of cytoplasmic *β*-catenin accumulation in our study. This was shown by direct sequencing of the coding regions of APC and *β*-catenin and by an APC protein truncation test. The mechanism that causes cytoplasmic stabilization in gliomas, therefore, seems to be located upstream of the *β*-catenin degradation complex and might relate to downregulation of *wnt* pathway inhibitors or a shift of *β*-catenin from the membranous compartment to the cytoplasm by tyrosine phosphorylation of *β*-catenin. EGFR [24], for instance, is frequently activated in high-grade gliomas and comprises a receptor tyrosine kinase capable of phosphorylating *β*-catenin and thus activating *wnt* signalling. Additionally, signalling pathways that are commonly disinhibited in gliomas including the TGF*β*/Akt and HGF pathway are also known to influence *wnt* signalling [2–4]. Furthermore, recent data indicate that several genes encoding upstream inhibitors of *wnt* signalling, such as SFRP1, SFRP2, and NKD2, are often epigenetically silenced by promoter hypermethylation in glioblastomas of WHO grade IV and that these alterations occur independently of common *β*-catenin mutations [25].

For various human cancers, an association between aberrant *wnt* pathway activation and the clinical behavior of the respective tumor is well established. In human breast cancer, cytoplasmic and nuclear localization of *β*-catenin were shown to predict a poor outcome [26, 27] whereas nuclear immunoreactivity of *β*-catenin is established as a favorable-risk indicator in a subgroup of medulloblastomas [28]. In human gliomas, there is to date little information about the clinical relevance of *wnt* signalling. Here we report on a WHO grade-dependent increase in *β*-catenin and decrease in the inactivated *β*-catenin-P654 in a large study sample. *β*-catenin-P654 expression even was significantly associated with prolonged OS. These results are supported by a recent study that demonstrated a significant increase of *β*-catenin mRNA expression levels in high-grade astrocytomas compared to low-grade astrocytomas and normal brain tissue [29]. Finally, we describe a significant correlation between the expression of *β*-catenin and the neural stemcell-associated protein nestin. Although our study suggests that *β*-catenin is not directly involved in the transcriptional regulation of nestin, *wnt*/*β*-catenin signalling could be involved in maintaining an undifferentiated, stem-like glioma cell phenotype. Consistently, the canonical *wnt* pathway has been reported to modulate the balance between stemness and differentiation in several adult stem cell niches [30] including neural progenitor cells in the adult brain [31–33]. 

Taken together, our results imply that aberrant expression of *β*-catenin in astrocytic gliomas is linked to a higher tumor grade and coincides with the expression of the stemness marker nestin. However, an impact on patient outcome was especially observed in low-grade gliomas.

## Figures and Tables

**Table 1:** Primer sequences used for PCR.

Target	Forward primer	Reverse primer
*β*-Catenin	5^′^-AGCGTGGACAATGGCTACTCAA-3^′^	5^′^-AAACATAGCAGCTCGTACCCTCT-3^′^
APC	5^′^-AGGACATG TTCTATGCCT TATGCC-3^′^	5^′^-CTTGGCATTAGATGAAGGTGTGGA-3^′^
GSK-3*β*	5^′^-ATCGGGATATTAAACCGCAGAACC-3^′^	5^′^-CTGTGTAGTTTGGGTTCATTTCTCT-3^′^
TCF1	5^′^-CCTCTCTGGCTTCTACTCCCT-3^′^	5^′^-CAGCCTGGGTATAGCTGCATGT-3^′^
TCF4	5^′^-AGGCACAGCTGTTTGGTCTAGAA-3^′^	5^′^-TCTCAGGGCCACGCCATCTTCA-3^′^
APC segment 1 codons 2–812	5^′^-GGATCCTAATACGACTCACTATAGGAACAGACCACCATG GCTGCAGCTTCATATGATC-3^′^	5^′^-CTGACCTATTATCATCATGTCG-3^′^
APC segment 2 codons 654–1225	5^′^-GGATCCTAATACGACTCACTATAGGAACAGACCACCATG CAAATCCTAAGAGAGAACAACT-3^′^	5^′^-GAGGATC CATTAGATGAAGGTGTGGACG-3^′^
APC segment 3 codons 1050–1693	5^′^-GGATCCTAATACGACTCACTATAGGAACAGACCACCATG GCAAGACCCAAACACATAATAG-3^′^	5^′^-GAGGATC CTGTAGGAATGGTATCTCG-3^′^
APC segment 4 codons 1582–2337	5^′^-GGATCCTAATACGACTCACTATAGGAACAGACCACCATG GCCATGCCAACAAAGTCATCA-3^′^	5^′^-CTTATTCCATTTCTACCAGGGGAA-3^′^
APC segment 5 codons 2123–2698	5^′^-GGATCCTAATACGACTCACTATAGGAACAGACCACCATGGGTTTATCTAGACAAGCTTCG-3^′^	5^′^-TTGAATCTTTAATGTTTGGATTTGC-3^′^
APC *β*-catenin binding region	5^′^-AGGACATGTTCTATGCCTTATGCC-3^′^	5^′^-CTTGGCATTAGATGAAGGTGTGGA-3^′^
APC mutational hot spot at codon 1309	5^′^-AACGTCATGTGGATCAGCCTATTG-3^′^	5^′^-GCTGGCAATCGAACGACTCTCAA-3^′^
APC mutational cluster region	5^′^-TTTAGCAGATGTACTTCTGTCAGTT-3^′^	5^′^-ATAGGTCCTTTTCAGAATCAATAGT-3^′^
*β*-catenin exon3	5^′^-CCAATCTACTAATGCTAATACTG-3^′^	5^′^-CTGCATTCTGACTTTCAGTAAGG-3^′^
GAPDH	5^′^-GGTGAAGGTCGGAGTCAACGGA-3^′^	5^′^-GAGGGATCTCGCTCCTGGAGGA-3^′^

**Table 2:** Clinicopathological characteristics of patients included in TMA analysis.

Histological diagnosis		Patients	
		*n* = 283 (%)	
Glioblastoma	WHO IV	221 (78.1)	
Anaplastic astrocytoma	WHO III	17 (6.0)	
Diffuse astrocytoma	WHO II	45 (15.9)	

Age		mean (*y*) ± SD	

Glioblastoma	WHO IV	53.8 ± 12.8	
Anaplastic astrocytoma	WHO III	36.1 ± 14.2	
Diffuse astrocytoma	WHO II	33.6 ± 18.7	

Gender		M : F	

Glioblastoma	WHO IV	133 : 88	
Anaplastic astrocytoma	WHO III	11 : 6	
Diffuse astrocytoma	WHO II	30 : 15	

Extent of resection		Total	Subtotal/biopsy
		*n* = (%)	*n* = (%)

Glioblastoma	WHO IV	151 (68.3)	70 (31.7)
Anaplastic astrocytoma	WHO III	11 (64.7)	6 (35.3)
Diffuse astrocytoma	WHO II	33 (73.3)	12 (26.7)

Adjuvant treatment^†^		Radiotherapy	Chemotherapy
		*n* = (%)	*n* = (%)

Glioblastoma	WHO IV	186 (84.2)	87 (39.4)
Anaplastic astrocytoma	WHO III	16 (94.1)	1 (5.9)
Diffuse astrocytoma	WHO II	14 (31.1)	3 (6.7)

^†^Therapy at primary tumour diagnosis.

**Table 3:** Results from multivariate survival analysis. Hazard ratios were adjusted for WHO grade, age at diagnosis, and extent of tumour resection.

Antigen expression	HR (95% CI)	*P* value
*β*-Catenin		

In WHO grade II–IV (≤50% versus >50% positive cells)	1.49 (0.83–2.69)	0.18
In WHO grade II (≤50% versus >50% positive cells)	Not determined	Not determined
In WHO grade III (≤50% versus >50% positive cells)	Not determined	Not determined
In WHO grade IV (≤50% versus >50% positive cells)	1.63 (0.90–2.95)	0.10

*β*-Catenin-P654		

In WHO grade II–IV (≤5% versus >5% positive cells)	0.85 (0.63–1.14)	0.27
In WHO grade II (≤5% versus >5% positive cells)	0.37 (0.14–0.96)	0.04
In WHO grade III (≤5% versus >5% positive cells)	0.65 (0.10–4.04)	0.64
In WHO grade IV (≤5% versus >5% positive cells)	0.96 (0.70–1.32)	0.81
